# Genome-wide analysis of anorexia nervosa and major psychiatric disorders and related traits reveals genetic overlap and identifies novel risk loci for anorexia nervosa

**DOI:** 10.1038/s41398-023-02585-1

**Published:** 2023-09-01

**Authors:** Lasse Bang, Shahram Bahrami, Guy Hindley, Olav B. Smeland, Linn Rødevand, Piotr P. Jaholkowski, Alexey Shadrin, Kevin S. O’ Connell, Oleksandr Frei, Aihua Lin, Zillur Rahman, Weiqiu Cheng, Nadine Parker, Chun C. Fan, Anders M. Dale, Srdjan Djurovic, Cynthia M. Bulik, Ole A. Andreassen

**Affiliations:** 1 Department of Child Health and Development, Norwegian Institute of Public Health, Oslo, Norway; 2 Regional Department for Eating Disorders, Division of Mental Health and Addiction, Oslo University Hospital, Oslo, Norway; 3 NORMENT Centre, Institute of Clinical Medicine, University of Oslo and Division of Mental Health and Addiction, Oslo University Hospital, Oslo, Norway; 4 Institute of Psychiatry, Psychology and Neuroscience, King’s College London, London, UK; 5 Department of Radiology, University of California, San Diego, La Jolla, CA USA; 6 Department of Cognitive Science, University of California, San Diego, La Jolla, CA USA; 7 Multimodal Imaging Laboratory, University of California San Diego, La Jolla, CA USA; 8 Department of Psychiatry, University of California, San Diego, La Jolla, CA USA; 9 Department of Neurosciences, University of California San Diego, La Jolla, CA USA; 10 Department of Medical Genetics, Oslo University Hospital, Oslo, Norway; 11 NORMENT Centre, Department of Clinical Science, University of Bergen, Bergen, Norway; 12 Department of Psychiatry, University of North Carolina at Chapel Hill, Chapel Hill, NC USA; 13 Department of Medical Epidemiology and Biostatistics, Karolinska Institutet, Stockholm, Sweden; 14 Department of Nutrition, University of North Carolina at Chapel Hill, Chapel Hill, NC USA

**Keywords:** Medical genetics, Psychiatric disorders

## Abstract

Anorexia nervosa (AN) is a heritable eating disorder (50–60%) with an array of commonly comorbid psychiatric disorders and related traits. Although significant genetic correlations between AN and psychiatric disorders and related traits have been reported, their shared genetic architecture is largely understudied. We investigated the shared genetic architecture of AN and schizophrenia (SCZ), bipolar disorder (BIP), major depression (MD), mood instability (Mood), neuroticism (NEUR), and intelligence (INT). We applied the conditional false discovery rate (FDR) method to identify novel risk loci for AN, and conjunctional FDR to identify loci shared between AN and related phenotypes, to summarize statistics from relevant genome-wide association studies (GWAS). Individual GWAS samples varied from 72,517 to 420,879 participants. Using conditional FDR we identified 58 novel AN loci. Furthermore, we identified 38 unique loci shared between AN and major psychiatric disorders (SCZ, BIP, and MD) and 45 between AN and psychological traits (Mood, NEUR, and INT). In line with genetic correlations, the majority of shared loci showed concordant effect directions. Functional analyses revealed that the shared loci are involved in 65 unique pathways, several of which overlapped across analyses, including the “signal by MST1” pathway involved in Hippo signaling. In conclusion, we demonstrated genetic overlap between AN and major psychiatric disorders and related traits, and identified novel risk loci for AN by leveraging this overlap. Our results indicate that some shared characteristics between AN and related disorders and traits may have genetic underpinnings.

## Introduction

Anorexia nervosa (AN) is an eating disorder characterized by restricted energy intake, abnormally low body weight, intense fear of weight-gain, and body image disturbances [1]. The age of onset is typically in early adolescence and the disorder disproportionally affects females. The estimated lifetime prevalence is 0.62% among females and 0.04% among males [2], but prevalence has increased in recent decades [3], particularly during the Covid-19 pandemic [4, 5]. Medical complications due to malnutrition and low body weight are common [6] and mortality rates are high [7]. Evidence of effective psychological and pharmacological treatments is limited [8], and treatment outcomes are often unsatisfactory [8, 9].

Although its core clinical characteristics relate to eating and body image disturbances, a variety of comorbid psychopathologies and related traits are commonly associated with AN. Patients tend to be characterized by neuroticism and perfectionism, and commonly present with anxious personality disorders [10, 11]. AN also often co-occurs with alexithymia, which involves difficulties in identifying and describing emotions [12]. Other commonly comorbid psychiatric conditions include mood and anxiety disorders [9, 13, 14], most notably major depression (MD). Evidence of psychotic features has also been reported in AN, with patients at increased risk of schizophrenia (SCZ) [15] and exhibiting delusional thoughts in relation to body image [15–17]. Moreover, AN is associated with cognitive traits such as increased intelligence [18]. Understanding the genetic underpinnings of the association between AN and the abovementioned major psychiatric disorders and related traits may provide insights into the genetic architecture of AN and its comorbidities.

Genetic variation substantially influences risk for AN, with twin-based heritability estimates of 50–60% [19]. Recent genome-wide association studies (GWAS) have identified the first genetic risk loci for AN [20, 21] and highlighted significant positive genetic correlations between AN and other psychiatric disorders, including obsessive-compulsive disorder, SCZ, MD, and bipolar disorder (BIP) [21–23]. Furthermore, studies have documented familial associations between AN and MD [24–26], SCZ [15, 26], and related disorders [27]; indicating shared familial liability factors. These findings align with converging evidence of shared genetic risk across multiple distinct psychiatric disorders [22], underscoring the complexity of genetic relationships among psychiatric phenotypes, including AN. Despite these advances, the genetic architecture of the disorder is largely unknown, and few studies have identified the shared risk loci between AN and other mental disorders. Characterizing the shared genetic architecture between AN and related phenotypes is a promising avenue for insight into genetic liability for AN itself, to inform nosology, guide research into pathophysiological mechanisms and suggest novel pharmacological treatments [28].

In the current study, we sought to identify novel risk loci for AN and investigate the shared genetic architecture of AN and related phenotypes including SCZ, BIP, MD, mood instability (Mood), neuroticism (NEUR), and intelligence (INT). Specifically, we leveraged the boost in statistical power by combining two GWAS to identify AN loci conditioned on their association with other phenotypes, using pleiotropy-based conditional false discovery rate (FDR) statistics [29–31]. Further, we applied conjunctional FDR to identify shared genetic loci, thereby revealing additional shared polygenic architecture between AN and related psychiatric disorders and traits.

## Methods

### Genome-wide association samples

We used GWAS summary statistics for AN [21], SCZ [32], BIP [33], and MD [34]; from the Psychiatric Genomics Consortium. The AN sample included 16,992 individuals with AN and 55,525 controls [21]. The SCZ sample included 40,675 individuals with SCZ and 64,643 controls [32]. The BIP sample consisted of 40,463 individuals with BIP and 313,436 controls [33]. The MD sample consisted of 105,718 individuals with MD and 344,901 controls [34]. To avoid sample overlap, we excluded the UK Biobank cohort from our MD and BIP samples. Additionally, we used GWAS summary statistic results for the following psychological traits: Mood [35], NEUR [36], and INT [37]. The Mood sample included 363,705 individuals; the NEUR sample included 372,903 individuals; and the INT sample included 269,867 individuals. The Supplementary information provides a brief account of the phenotype definitions for each GWAS sample, and additional details are provided in the original GWAS studies [21, 32–37].

### Statistical analyses

We estimated genetic correlations between AN and each disorder and psychological trait using linkage disequilibrium (LD) score regression [38, 39]. To assess the presence of cross-phenotype polygenic enrichment, we generated conditional quantile-quantile (Q-Q) plots [29], conditioning AN on each disorder (SCZ, BIP, MD) and psychological trait (Mood, NEUR, and INT), and vice versa. We compared the enrichment of associations of all single nucleotide polymorphisms (SNPs) with SNPs associated with the conditional trait (i.e., the major psychiatric disorders and psychological traits), at increasingly significant *p*-value thresholds (0.1, 0.01, and 0.001). Successive deflections of the Q-Q plot away from the null line with increasing strength of association with the conditional trait is indicative of cross-phenotype polygenic enrichment.

Next, we employed the conditional FDR (condFDR) method [29, 31] which leverages cross-phenotype enrichment observed on the conditional Q-Q plots to improve the discovery of genetic loci associated with AN. This method builds on an empirical Bayesian statistical framework, using GWAS summary statistics from a primary trait of interest (e.g., AN) together with those of a conditional trait (e.g., SCZ) to estimate the posterior probability that a SNP has no association with the primary trait, provided that the *p* values for that SNP in both the primary and conditional traits are as small as or smaller than the observed *p* value. The condFDR method increases the power to identify genetic variants associated with the primary trait of interest by reranking the test statistics of the primary trait based on the strength of association with the conditional trait.

To determine shared genetic loci between AN and related psychiatric disorders and psychological traits we employed the conjunctional FDR (conjFDR) method [28–30]. This method is defined as the maximum of the two condFDR statistics for a specific SNP, i.e. for trait A conditional on trait B and trait B conditional on trait A. This represents an estimate for the posterior probability that a SNP is null for either or both traits, provided that the *p* values for both phenotypes are as small as or smaller than the *p* values for each trait individually. More details are found in the original [29] and subsequent publications [31].

In the current study, we used an FDR level of 0.05 per pairwise comparison for cond/conjFDR. Manhattan plots based on the conjFDR were generated to depict the genomic location of shared genetic loci [29]. All analyses were performed after excluding SNPs in the extended major histocompatibility complex (MHC; hg19 location chr 6: 25119106-33854733) and the 8p23.1 (hg19 location chr 8: 7242715-12483982) regions to avoid potential biases due to complex LD.

### Genomic loci definition

We used the FUMA protocol to define independent genomic loci jointly associated with AN and each psychiatric disorder and psychological trait [40]. The genomic loci were defined based on the lead SNPs and candidate SNPs within each locus as significant SNPs within a LD *r*^*2*^ >= 0.6 and cond/conjFDR <0.1 with at least one related independent significant SNP. Independent significant SNPs were considered as cond/conjFDR < 0.05 and *r*^*2*^ < 0.6 while lead SNPs were defined if they were in approximate linkage equilibrium with each other (*r*^*2*^ < 0.1). If two or more lead SNPs located within one LD block (in 250 kb), we merged them into one genomic risk locus. LD information from the European population was derived from the 1000 Genomes Project reference panel [41]. We used Bedtools [42] to identify shared loci across pair-wise analyses and to identify unique genetic loci across all analyses. Novel loci were determined by cross-referencing identified loci with previous GWASs and other relevant studies.

### Functional annotation

We used FUMA to annotate identified shared variants according to function, the predicted deleteriousness (Combined Annotation Dependent Depletion score, [43], regulatory effect (RegulomeDB scores, [44] and chromatin states. We next mapped genes to candidate SNPs using three gene-mapping methodologies using FUMA: 1) positional mapping which matches SNPs by physical position to all genes within a 10 kb window 2) expression quantitative trait loci (eQTL) mapping that identifies genes whose expression is associated with the SNPs’ allelic variation, 3) chromatin interaction mapping that matches SNPs to genes with which they are predicted to interact based on chromatin structure [40]. We applied gene-set analyses (on all genes) using FUMA [45, 46], pathway analyses using Consensus PathDB [47] and spatiotemporal gene expression analysis of mapped genes in the R package “cerebroViz” using BrainSpan RNA sequencing data [48, 49]. All analyses were corrected for multiple comparisons using Bonferroni correction.

## Results

### Genetic overlap and correlation between AN and related psychiatric disorders and psychological traits

**Fig. 1:**
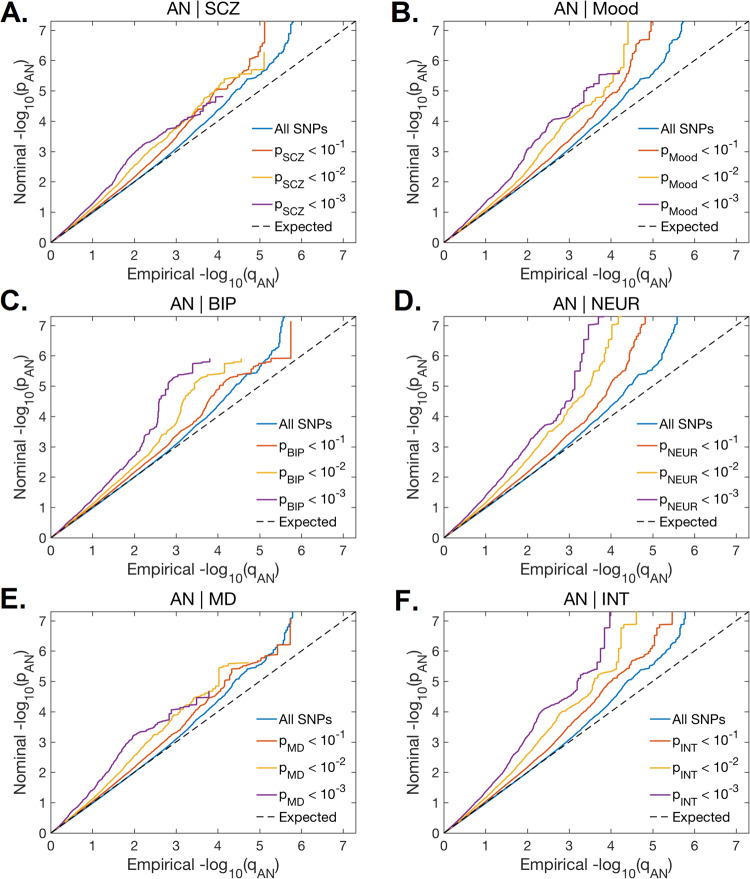
Panels A-F show conditional quantile-quantile plots of nominal vs. empirical anorexia nervosa (AN) -log_10_ *p*-values as a function of the significance of the association with schizophrenia (SCZ), bipolar disorder (BIP), major depression (MD), mood instability (Mood), neuroticism (NEUR), and intelligence (INT) at the level of *p* < .10, *p* < .01 and *p* < .001. These plots show the quantiles of the observed *p*-values on the y-axis against the theoretical quantiles under no association on the x-axis. Deflections from the null line indicate systematic association.

The conditional Q-Q plots showed successive increments of SNP enrichment for AN conditioned on association *p-*values for each psychiatric disorder and psychological trait (Fig. 1). The consistently increasing leftward deflection for subsets of variants with higher significance in the conditional trait in both directions indicates substantial polygenic overlap between AN and the other psychiatric disorders and related traits. See Supplemental Figure S1 for the reverse Q-Q plots.

Genome-wide LD score regression showed significant positive genetic correlations between AN and the following disorders and traits: SCZ (*r*_g_ = 0.26, SE = 0.03, *p* = 6.64 × 10^−14^), BIP (*r*_g_ = 0.19, SE = 0.04, *p* = 1.18 × 10^−6^), MD (*r*_g_ = 0.35, SE = 0.06, *p* = 1.59 ×10^−9^), NEUR (*r*_g_ = 0.26, SE = 0.04, *p* = 1.52 × 10^−12^), and INT (*r*_g_ = 0.10, SE = 0.04, *p* = 3.80 × 10^−3^). Genetic correlation between AN and Mood were not significant (*r*_g_ = 0.06, SE = 0.05, *p* = 0.22). Please note that the statistical significance of certain genetic correlations (e.g., AN and SCZ) is higher than others (e.g., MD and SCZ) despite a lower point estimate because the precision of the estimate is greater. This is likely due to the higher heritability and discoverability of certain phenotypes (e.g., higher for SCZ compared to MD).

### AN-associated loci identified with condFDR

Using condFDR analysis (FDR < 0.05), we identified 40, 37, and 49 loci associated with AN conditionally on SCZ, BIP, and MD, respectively (Supplementary Tables 1–3). For the psychological traits, we identified 48, 55, and 60 loci associated with AN conditionally on Mood, NEUR, and INT, respectively (Supplementary Tables 4–6). Across all conditional analyses, there were 58 unique loci associated with AN, of which all were novel.

### Loci shared between AN and related psychiatric disorders and psychological traits

**Fig. 2:**
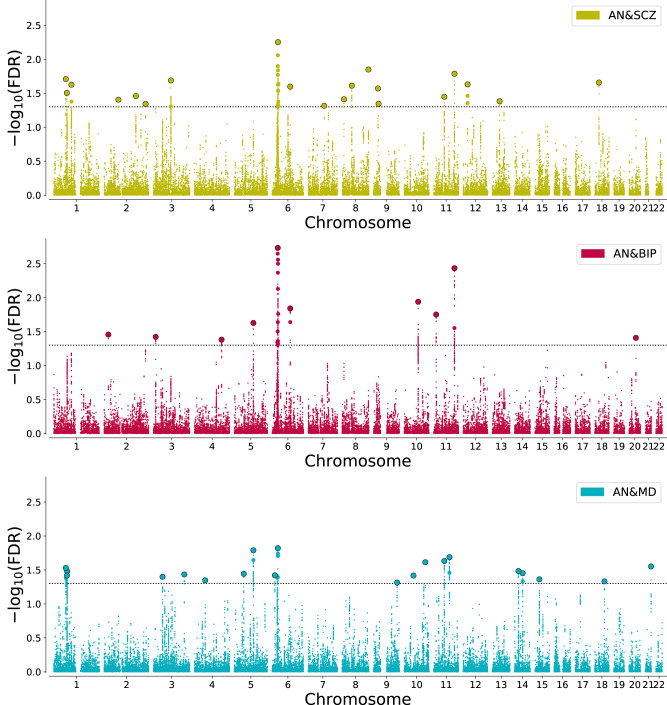
Manhattan plots based on the -log_10_ transformed conjFDR values for each single-nucleotide polymorphism, depicting common genetic variants shared between anorexia nervosa (AN), schizophrenia (SCZ), bipolar disorder (BIP), and major depression (MD). The dotted horizontal line represents the threshold chosen for reporting shared associations (conjFDR < 0.05). Circles indicate lead SNPs.

**Fig. 3:**
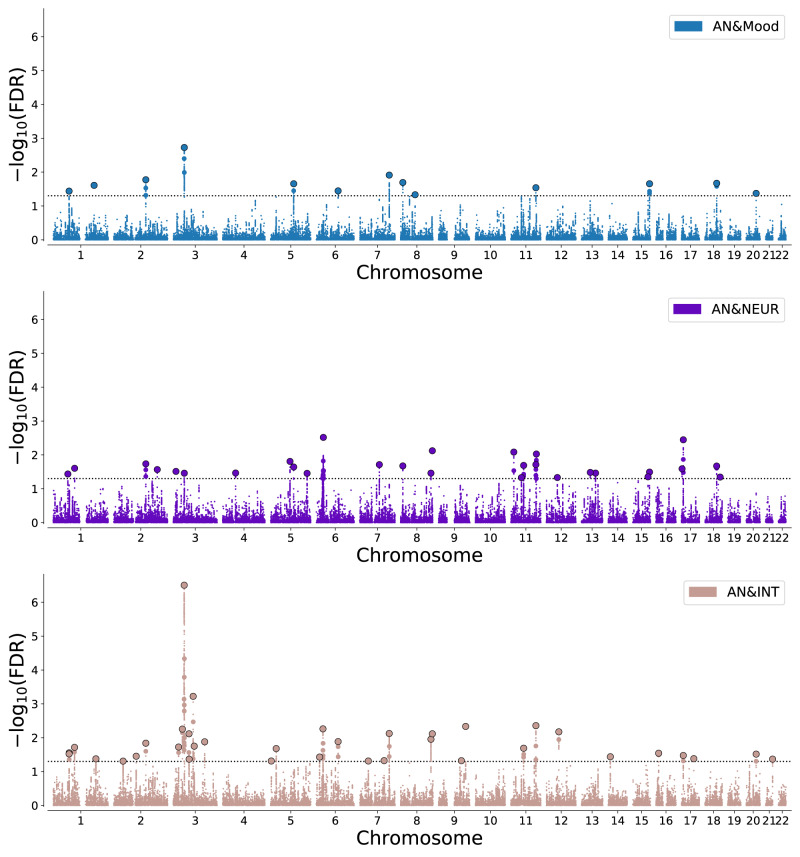
Manhattan plots based on the -log_10_ transformed conjFDR values for each single-nucleotide polymorphism, depicting common genetic variants shared between anorexia nervosa (AN), mood instability (Mood), neuroticism (NEUR), and intelligence (INT). The dotted horizontal line represents the threshold chosen for reporting shared associations (conjFDR < 0.05). Circles indicate lead SNPs.

ConjFDR analysis revealed numerous loci shared between AN and the related disorders and traits; see Table 1 and Supplementary Tables 7–12. Specifically, 20, 10, and 20 loci were jointly associated with SCZ, BIP, and MD, respectively. In total, we identified 38 unique shared loci between AN and related psychiatric disorders. Furthermore, 13, 29, and 36 loci were jointly associated with Mood, NEUR, and INT, respectively. In total, 45 loci were uniquely shared between AN and the psychological traits; of which 9 overlapped with the 38 uniquely shared between AN and psychiatric disorders. After merging physically overlapping condFDR loci across all analyses, there were 56 “unique” loci associated with AN. See Figs. 2–3 for Manhattan plots of all shared loci.

**Table 1:** Results of conjunctional FDR showing number of shared loci between AN and related disorders and psychological traits.

Secondary phenotype	Secondary GWAS (*n/n*_*effective*_ *for case-control GWASs*)^1^	Shared loci with AN at conjFDR < 0.05	Novel loci in AN (*n*)	Novel loci in comparison phenotype (*n*)
SCZ	126,282	20	18	4
BIP	150,670	10	10	3
MD	389,039	20	19	9
Mood	356,933	13	12	4
NEUR	372,903	29	26	7
INT	269,867	36	31	16

*Note:* *AN* Anorexia nervosa, *BIP* Bipolar disorder, *conjFDR* Conjunctional False Discovery Rate, *INT* Intelligence, *Mood* Mood instability, *NEUR* Neuroticism, *SCZ* Schizophrenia. ^1^*n*_*effective*_ = 4/(1/*n*_*cases* +_ 1/*n*_*controls*_).

A pattern of concordant genetic effects was evident when comparing the effect directions of lead SNPs. Concordant effect directions were observed for 19 of 20 (95%) shared loci in AN and SCZ, 7 of 10 (70%) shared loci in AN and BIP, and 18 of 20 (90%) shared loci in AN and MD (Supplementary Tables 7–9). For the psychological traits, concordant association directions were observed for 8 of 13 (62%) shared loci in AN and Mood, 28 of 29 (96%) shared loci in AN and NEUR, and 21 of 36 (58%) shared loci in AN and INT (Supplementary Tables 10–12).

### Annotation of loci shared between AN and related psychiatric disorders and psychological traits

We functionally annotated all SNPs in LD (*r*^2^ ≥ 0.6) with a significant independent SNP and with conjFDR <0.1 within the shared loci associated with AN and related disorders and traits using FUMA. The majority of SNPs jointly associated with AN and SCZ, BIP, Mood and INT were intronic; while the majority of SNPs jointly associated with AN and MD and NEUR were intergenic. For more details see Supplementary information, Figure S2 and Supplementary Tables 7–12).

We mapped candidate SNPs to genes by three independent gene-mapping strategies. In total 187 unique protein coding genes, including 125, 115, and 109 genes were mapped between AN and SCZ, BIP and MD, respectively. Eighty of these genes were mapped by at least two out of the three gene mapping strategies. Gene set analysis of these genes did not show any overrepresented gene-ontology (GO) terms after excluding MHC region from the analysis. Similarly, 294 unique protein coding genes were mapped to the candidate SNPs in the shared loci across AN and psychological traits, including 165, 204 and 53 mapped across AN and INT, NEUR and Mood, respectively. The gene set analysis showed that the mapped genes were overrepresented in 223 GO terms for AN and psychological traits. See Supplementary Table 37 for all GO gene sets. The most significantly overrepresented gene-set was “synapse organization” (*p* = 2.87 × 10^−7^), followed by “negative regulation of intracellular steroid hormone receptor signaling pathway” (*p* = 1.16 × 10^−6^), “regulation of transport” and “neuron development”.

Among genes mapped to loci shared between AN and psychiatric disorders, several immune-related genes were implicated, including the C4A, C4B, NCAM1, HLA-B, HLA-C, and HLA-E genes. Both the C4A [50, 51] and C4B [51] genes have previously been associated with the etiology of SCZ and BIP. The C4A, NACM1, HLA-B, HLA-C, and HLA-E genes were also mapped to loci shared between AN and NEUR, and AN and INT. This suggests genes associated with AN risk are also associated with a range of other disorder- and trait-related phenotypes. However, genes mapped to the MHC region must be interpreted with caution due to the complex LD in this genomic region. See Table 2 for an overview of genes mapped to lead SNPs within loci jointly associated with anorexia nervosa and at least two related phenotypes (see Supplementary Tables 25–30 for full list of genes).

**Table 2:** Genes mapped to lead single nucleotide polymorphisms within loci jointly associated with anorexia nervosa and at least two related phenotypes.

Phenotypes	Symbol	Chr	Phenotypes	Symbol	Chr
MDD, INT	ADARB1	21	Mood, NEUR	LINC00923	15
MDD, INT	AMT	3	Mood, INT	LRGUK	7
Mood, NEUR, INT	ARHGAP15	2	SCZ, BIP, MDD, NEUR	MICB	6
BIP, NEUR	ARNTL	11	SCZ, NEUR	MSH5	6
BIP, MDD	BAG6	6	SCZ, Mood, NEUR	MSRA	8
SCZ, NEUR	C2	6	BIP, Mood, NEUR, INT	NCAM1	11
SCZ, BIP, MDD, NEUR	C4A	6	MDD, INT	NCKIPSD	3
MDD, INT	CCDC71	3	SCZ, MDD, Mood, INT	NEGR1	1
SCZ, BIP, MDD, NEUR	CCHCR1	6	Mood, INT	NEURL2	20
SCZ, NEUR	CFB	6	MDD, NEUR, INT	OR5AK2	11
SCZ, NEUR	CSNK2B	6	MDD, NEUR	P4HTM	3
Mood, INT	CTSA	20	Mood, INT	PCIF1	20
SCZ, BIP, MDD, NEUR	DDAH2	6	SCZ, MDD, NEUR	PDE4B	1
MDD, INT	DTNBP1	6	Mood, NEUR	RBM6	3
Mood, NEUR	ERI1	8	Mood, NEUR	RNF123	3
Mood, INT	EXOC4	7	Mood, INT	SPATA25	20
Mood, NEUR	FAM212A	3	MDD, Mood, NEUR	TCF4	18
BIP, MDD	FLOT1	6	BIP, NEUR	TIMP4	3
MDD, Mood, NEUR	GPX1	3	BIP, INT	TRIM26	6
BIP, MDD	HLA-B	6	SCZ, NEUR, INT	TSNARE1	8
SCZ, BIP, MDD, NEUR	HLA-C	6	SCZ, BIP, MDD, NEUR, INT	VARS2	6
SCZ, NEUR	HLA-DQA1	6	MDD, INT	WDR6	3
SCZ, NEUR	HLA-DQB1	6	Mood, INT	ZSWIM1	20

*Note:* Genes mapped to lead SNPs within loci jointly associated with AN and at least two related phenotypes (SCZ, BIP, MD, Mood, NEUR, and INT) at conjFDR < 0.05. Genes shared between AN and related phenotypes mapped to genes using positional mapping, eQTL mapping, and chromatin interaction strategies. AN Anorexia nervosa, BIP Bipolar disorder, conjFDR Conjunctional False Discovery Rate, INT Intelligence, Mood Mood instability, NEUR Neuroticism, SCZ Schizophrenia.

Spatio-temporal analysis indicated that the expression of mapped genes to the shared loci between AN and psychiatric disorders (SCZ, BIP, and MD) decreased after middle childhood (Figure S2). The mapped genes to the shared loci between AN and Mood, and AN and NEUR were weakly expressed after late childhood, while the mapped genes to the shared loci between AN and INT were highly expressed from late childhood to late adolescence (Figure S3). A more detailed description of functional annotation analyses for all analyses and for each individual analysis is presented in Supplementary information.

### Pathway analysis of genes mapped to loci shared between AN and related psychiatric disorders and psychological traits

After excluding MHC regions; 1, 2, and 10 pathways were enriched for genes linked to the candidate SNPs shared between AN and SCZ, BIP and MD, respectively. None of these pathways were identified across all three analyses. Furthermore, 15, 14, and 35 pathways were enriched for genes linked to the candidate SNPs shared between AN and Mood, NEUR, and INT, respectively. Several of these were shared across multiple analyses, including the “Signaling by MST1”, “amb2 integrin signaling”, “pathways regulating Hippo signaling”, and “rho-selective guanine exchange factor akap13 mediates stress fiber formation” pathways. See Supplemental Tables 31–36 for all overrepresented pathways.

Based on unique genes mapped to the shared loci between AN and the psychiatric disorders (SCZ, BIP, and MD) we identified 7 significantly overrepresented pathways (see Supplemental Table 38). No new pathways emerged in this analysis; all pathways were also enriched for genes linked to the candidate SNPs shared between AN and MD. For the unique genes mapped to the shared loci between AN and the psychological traits (Mood, NEUR, and INT) we identified 36 significantly overrepresented pathways (see Supplemental Table 39). Most of these were also identified in individual analyses of Mood, NEUR, and INT. However, some additional pathways emerged, including “transcriptional regulation of granulopoiesis”, “EPHA forward signaling”, and “signaling by TGF-β receptor complex”.

## Discussion

In the current study, we demonstrated genetic overlap between AN and major psychiatric disorders and psychological traits, and identified novel risk loci for AN by leveraging this overlap. Our findings suggest a large degree of genetic overlap between AN and related psychiatric disorders and traits, possibly indicating overlapping genetic mechanisms [31]. Our findings shed light on the genetic etiology of AN, and could have clinical utility in developing risk prediction models and psychopharmacological treatments.

We identified 38 unique loci jointly associated with AN and psychiatric disorders, while 45 unique loci were jointly associated with AN and psychological traits. Nine of these loci overlapped. The effect direction of the lead SNPs on AN and each psychiatric disorder and related psychological traits revealed that the majority of lead SNPs had concordant effects, but to different degrees. Most lead SNPs had concordant effects on AN and each psychiatric disorder (70–95%), including 95% of loci shared with SCZ. Effect directions were also mostly concordant for the psychological traits (58–96%), the highest of which was for NEUR. These findings are in line with the positive genetic correlations between AN and MD, SCZ, BIP, and NEUR identified in the current and previous studies [20–22, 38]. Of note, the genetic correlation between AN and Mood was non-significant in our study, and our observed significant correlation between AN and BIP contrasts with findings from an earlier study based on smaller GWAS samples [38].

The genetic overlap between AN and MD aligns with epidemiological evidence of associations between these two disorders. MD is among the disorders most frequently comorbid with AN [9, 13, 14]. Both AN and MD are characterized by mood disturbances, and studies have shown that such symptoms are central features among patients with AN [52, 53]. Family studies have shown that AN and MD tend to co-aggregate in families [24–26], suggesting the disorders may share familial liability factors. Our findings suggests that at least part of the co-occurrence of these disorders is due to shared genetic liabilities, mirroring results from twin studies [54, 55].

Our finding that AN shows genetic overlap with BIP and SCZ is intriguing and may reflect shared clinical features or vulnerabilities. There is evidence of psychotic features in AN [56], including the pervasive body image disturbances that may be delusional by nature [15–17]. Also, difficulty recognizing and describing feelings (alexithymia) is frequent in eating disorders [12] and is also reported in MD, BIP and SCZ [57, 58], possibly representing a shared vulnerability factor. Previous findings indicate familial co-aggregation of AN and SCZ [15, 26],—implicating a genetic basis for the association. A recent systematic review also highlighted that BIP and eating disorders are frequently comorbid [59]. It is possible that AN, SCZ, and BIP share genetic vulnerabilities that are only partly reflective of the clinical features of each disorder. Instead, the genetic overlap between these disorders may reflect general liabilities to multiple psychiatric disorders, akin to the suggested “p” factor [60]. These overlapping vulnerabilities may be reflected in the underlying genetic architecture.

Our study also demonstrated genetic overlap between AN and the psychological traits Mood, NEUR, and INT. The overlap with Mood and NEUR aligns with epidemiological and genetic evidence supporting an association between AN and psychiatric disorders characterized by mood disturbances and high anxiety [9, 13, 14]. Previous studies have shown that patients with AN are characterized by high levels of depressive and anxious features even following recovery [61, 62]. In support of this, one study found that NEUR was prospectively related to development of AN [63]. Additionally, we documented genetic overlap between AN and INT, which could be related to the increased intelligence associated with AN [18]. In line with this, a previous GWAS found significant positive genetic correlations between AN and years of education [21]. They also found positive genetic correlations between INT and AN when specific AN subtypes were considered. Importantly, our findings show that the genetic overlap between AN and related phenotypes is not limited to diagnostically defined psychopathology, but extends to trait phenotypes (e.g., neuroticism) that are shared among common psychiatric disorders and may underlie the high comorbidity.

By leveraging the genetic overlap between AN and the included psychiatric disorders and psychological traits, we identified risk loci associated with AN conditional on each of the phenotypes. Specifically, we identified 40, 37, and 49 loci associated with AN conditional on SCZ, BIP, and MD respectively. We also identified 60, 55, and 48 loci associated with AN conditional on Mood, NEUR, and INT respectively. Among these risk loci were the eight loci previously reported in a recent GWAS study of AN [21]. Across all analyses, 58 novel risk loci for AN were identified. These findings highlight the benefit of leveraging the polygenic overlap between psychiatric disorders to boost discovery of novel risk loci. Extending our analyses to other phenotypes holds potential in revealing more risk loci for AN.

The mapped genes for the shared loci included a number of genes related to the immune system, for example the C4A gene, which was implicated across multiple analyses. While no pathways were identified for loci shared between AN and psychiatric disorders, several overlapping pathways were identified for loci shared between AN and the psychological traits. In particular, the “Pathways Regulating Hippo Signaling”, “Wnt Signaling Pathway“ and “signaling by MST1” pathways were implicated for shared loci with both Mood and INT. Hippo signaling regulates stemness, cell proliferation and apoptosis and disruption of these systems has been associated with metabolic and neurodegenerative diseases, mirroring findings from the primary AN GWAS which highlighted associations with genes involved in metabolic dysregulation [21]. Further, recent research showed that genes within the Hippo pathway modulate key molecular and cellular processes that are involved in the pathophysiology of stress-related-psychiatric disorders [64]. In addition to this, various proteins of the Hippo signaling pathway are linked via Wnt-signaling and other pathways to stress-regulated signaling cascades [65], while overexpression of MST1 induces memory impairment via disturbances in the patterns of neural activities. This mechanism may therefore contribute to the genetic overlap observed between AN and INT [66].

Our study has limitations. As with all GWAS findings, it is challenging to identify the true ‘causal SNP’ from correlated SNPs due to LD. Therefore, experimental studies and improved fine-mapping strategies are needed to determine the true causal variants underlying the shared associations reported here and whether the same causal variants are involved in AN and the included phenotypes. We also note that sample size for the AN GWAS is still small, and future studies utilizing larger samples may elucidate more shared genetic loci. Furthermore, the AN GWAS sample lacks ethnic diversity, with samples confined to North-America, Europe, Australia and New Zealand. Finally, we note that a small proportion of our AN sample (4.5%; 768 AN cases and 3065 controls) was derived from the UK Biobank, which also contributed to the MOOD, NEUR and INT samples (see Supplementary information). Sample overlap may inflate the cond/conjFDR statistics resulting in an increase in false positives, although since UKB cases and controls represented <5% of the total AN, this is unlikely to significantly change our results.

In conclusion, we used conditional and conjunctional FDR approach to identify novel risk loci for AN and demonstrate genetic overlap between AN and related psychiatric disorders and psychological traits. This expands on previous findings of shared genetic risk between AN and related phenotypes, advancing our understanding of the genetic underpinnings of AN. This increases the understanding the genetic architecture of AN, which can form a platform for developing risk prediction models and novel psychopharmacological treatments.

### Supplementary information

Supplementary-material

Dataset 1

## Data Availability

Data supporting the findings of this study are openly available from an online repository or are available on request from study authors. All code is freely available at https://github.com/precimed and https://github.com/bulik/ldsc. Analyses were conducted in Python v3.5, Matlab R2020b, and R v3.6.3. Locus definition, functional annotation, and gene-set analysis were performed using FUMA (https://fuma.ctglab.nl/).
