# Supplementary material for: Genome-wide analysis of anorexia nervosa and major psychiatric disorders and related traits reveals genetic overlap and identifies novel risk loci for anorexia nervosa
Source: Transl Psychiatry. 2023 Sep 1;13:291. doi: 10.1038/s41398-023-02585-1 (PMC10474135; doi:10.1038/s41398-023-02585-1)
Supplement: Supplementary file 1 — Supplementary-material [file 41398_2023_2585_MOESM1_ESM.docx]

**SUPPLEMENTARY INFORMATION**

**Genome-wide analysis of anorexia nervosa and major psychiatric disorders and traits reveals genetic overlap and identifies novel risk loci for anorexia nervosa.**

Lasse Bang (Ph.D.)^1,2*^, Shahram Bahrami (Ph.D.)^3*^, Guy Hindley^3,4^, Olav B. Smeland (M.D. Ph.D.)^3^, Linn Rødevand (Ph.D.)^3^, Piotr P. Jaholkowski^3^, Alexey Shadrin (Ph.D.)^3^, Kevin S. O’Connell (Ph.D.)^3^, Oleksandr Frei (Ph.D.)^3^, Aihua Lin (Ph.D.)^3^, Zillur Rahman (Ph.D.)^3^, Weiqiu Cheng (Ph.D.)^3^, Nadine Parker (Ph.D.)^3^, Chun C. Fan (M.D.)^5,6^, Anders M. Dale (Ph.D.)^5,7,8,9^, Srdjan Djurovic (Ph.D.)^8,10,11^, Cynthia M. Bulik (Ph.D.)^12,13,14^, Ole A. Andreassen (M.D. Ph.D.)^3^

^1^Department of Child Health and Development, Norwegian Institute of Public Health, Oslo, Norway;

^2^Regional Department for Eating Disorders, Division of Mental Health and Addiction, Oslo University Hospital;

^3^NORMENT Centre, Institute of Clinical Medicine, University of Oslo and Division of Mental Health and Addiction, Oslo University Hospital, 0407 Oslo, Norway;

^4^Institute of Psychiatry, Psychology and Neuroscience, King’s College London, 16 De Crespigny Park, London, SE5 8AB, UK

^5^Department of Radiology, University of California, San Diego, La Jolla, CA 92093, United States of America;

^6^Department of Cognitive Science, University of California, San Diego, La Jolla, CA, USA;

^7^Multimodal Imaging Laboratory, University of California San Diego, La Jolla, CA 92093, United States of America;

^8^Department of Psychiatry, University of California, San Diego, La Jolla, CA, USA.

^9^Department of Neurosciences, University of California San Diego, La Jolla, CA 92093, United States of America.

^10^Department of Medical Genetics, Oslo University Hospital, Oslo, Norway.

^11^NORMENT Centre, Department of Clinical Science, University of Bergen, Bergen, Norway.

^12^Department of Nutrition, University of North Carolina at Chapel Hill, Chapel Hill, NC, USA.

^13^Department of Medical Epidemiology and Biostatistics, Karolinska Institutet, Stockholm, Sweden.

^14^Department of Psychiatry, University of North Carolina at Chapel Hill, Chapel Hill, NC, USA.

* Equal contribution

**Supplementary methods p. 3-4.**

**Supplementary results p. 5-6.**

**Figure S1 p. 7.**

**Figure S2 p. 8-9.**

**Figure S3 p. 10-11.**

**References p. 11-12.**

**SUPPLEMENTARY METHODS**

**Sample overlap**

A small proportion of our AN sample (4.5%; 768 AN cases and 3065 controls) was derived from the UK Biobank, which also contributed to the BIP, MOOD, NEUR and INT samples (see Supplementary material). Since we had access to sub-study data for BIP, we therefore excluded UKB samples from our BIP summary statistics.

We were unable to exclude samples that overlapped in the conjFDR analyses of the remaining phenotypes MOOD, NEUR and INT. In the MOOD sample, 363,705 participants were derived from the UK Biobank. The corresponding numbers for NEUR and INT were 372,903 and 195,653. We note that since only a small proportion of our AN sample was derived from the UK Biobank, the sample overlap with MOOD, NEUR and INT samples is very small.

**Phenotyping**

Details regarding the phenotype definitions in the individual GWAS samples are described in the original papers. For the major psychiatric disorders anorexia nervosa (AN), schizophrenia (SCZ), bipolar disorder (BIP) and major depression (MD); the phenotype definitions involved fulfilling lifetime diagnostic (DSM) criteria for the disorder. Information on how the diagnostic criteria were assessed are described in the original papers (5–8).

Mood instability (Mood) was measured with the self-report yes/no question: “does your mood often go up and down?” (1). Neuroticism (NEUR) was measured with self-report questions based on the Eysenck Personality Questionnaire Revised Short Form and Big Five Inventory (2). Intelligence (INT) was measured with diverse cognitive measures, the majority of which relate to fluid domains of cognition (3). Measures included: verbal and mathematical reasoning, letter-digit substitution, Stroop, verbal fluency, delayed recall, SON-R, logical, verbal, spatial and technical ability subtests, SAT test scores, WISC-III, Raven's progressive matrices, WISC-IV, CANTAB factor score, SRT-C factor score, MAB-II IQ score, WAIS IQ score, ICAR verbal reasoning test and processing speed tests.

**Genomic loci definition and functional annotation**

We defined independent genomic loci identified by conditional or conjFDR analysis using the FUMA protocol (9). All SNPs with a conjFDR value < 0.10 having an LD *r*^2^ ≧ 0.6 with one of the independent significant SNPs were functionally annotated using multiple tools, performed in FUMA including (a) Combined Annotation Dependent Depletion scores (CADD) (10), (b) RegulomeDB scores (11), and (c) minimum chromatin state (12,13). A CADD score above 12.37 shows an association of deleterious protein with outcomes (10). The RegulomeDB score indicates the regulatory functionality of SNPs based on expression quantitative trait loci and chromatin marks (11). The chromatin state indicates the accessibility of genomic regions using 15 categories, as predicted by ChromHMM based on 5 chromatin marks for 127 epigenomes (12,13). FUMA’s default parameters were used to outline the distinct genomic loci and their borders (9).

Next, we used three independent gene-mapping strategies to map the lead SNPs to genes; (9): a) positional mapping to align SNPs to genes based on their physical proximity, b) expression quantitative trait locus (eQTL) mapping to match cis-eQTL SNPs to genes whose expression is associated with allelic variation at the SNP level, and c) chromatin interaction mapping to link SNPs to genes based on three-dimensional DNA–DNA interactions between each SNP’s genomic region and nearby or distant genes. All gene mapping strategies were limited to brain tissues.

In addition, we applied spatio-temporal analysis to show patterns of gene expression of the genes mapped to SNPs in the shared loci between AN and the related disorders and traits across 11 brain tissues at 11 developmental timepoints (14–16). Finally, pathway over-representation analyses were performed for each of these mapped genes using ConsensusPathDB (17). All analyses were corrected for multiple comparisons, and significance set at a two-sided *p*-value < .05.

**SUPPLEMENTARY RESULTS**

**Annotation of loci shared between AN and related psychiatric disorders and traits**

We functionally annotated all SNPs in LD (*r*^2^ > 0.6) with a significant independent SNP and with conjFDR < 0.1 within the shared loci associated with AN and related disorders and traits (see Supplementary Tables 7-12). Of all SNPs jointly associated with AN and SCZ; 38.4% were intronic, 33.2% were intergenic, and 1.1% were exonic (see Figure S1). For the SNPs jointly associated with AN and BIP; 62.9% were intronic, 19.1% were intergenic and 1.8% were exonic (see Figure S1). For the SNPs jointly associated with AN and MD; 26.3% were intronic, 52.6% were intergenic, and 1.0% were exonic (see Figure S1). For the SNPs jointly associated with AN and mood; 70.6% were intronic, 3.9% were intergenic, and 2.1% were exonic (see Figure S1). For the SNPs jointly associated with AN and neuroticism; 30.5% were intronic, 43.4% were intergenic, and 1.2% were exonic (see Figure S1). Finally, for the SNPs jointly associated with AN and intelligence; 49.0% were intronic, 35.5% were intergenic, and 1.5% were exonic (see panel F in Figure S1).

Further results showed that 3.6% (*n* = 40) of the candidate SNPs jointly associated with AN and SCZ had CADD scores > 12.37, suggesting high deleteriousness and two of them had RegulomeDB scores of 1a to 1f, which may affect transcription factor binding (Supplementary Table 13). One of the lead SNP “rs7314161” had CADD = 13.43. For candidate SNPs jointly associated with AN and BIP, 5.5% (*n* = 42) showed high deleteriousness and three of them had low RegulomeDB (Supplementary Table 14). For candidate SNPs jointly associated with AN and MD, 3.9% (*n* = 47) showed similar high deleteriousness (Supplementary Table 15). Two of these SNPs were also lead SNPs “rs12629759” (with CADD = 15.24 and RegulomeDB = 1f) and rs2507989 (with CADD = 13.26). Of the candidate SNPs jointly associated with AN and mood, 5.6% (*n* = 40) had CADD scores > 12.37 and one of them was lead SNP “rs4430884” (Supplementary Table 16). For SNPs jointly associated with AN and neuroticism, 5.1% (*n* = 80) showed high deleteriousness and two of them had low RegulomeDB (Supplementary Table 17). Finally, 4.9% (*n* = 120) of the candidate SNPs jointly associated with AN and intelligence showed high deleteriousness and nine of them showed affect transcription factor binding with RegulomeDB 1f (Supplementary Table 18).

We next used three gene mapping strategies with FUMA to map protein-coding genes to annotated SNPs within the shared loci (see Supplementary Tables 25-30). Fourteen of the 20 loci jointly associated with AN and SCZ mapped to 29 genes (Supplementary Table 25). Seven of the 10 loci jointly associated with AN and BIP mapped to 38 genes (Supplementary Table 26). Twelve of the 20 jointly associated with AN and MD mapped to 36 genes (Supplementary Table 27). Eleven of the 13 loci jointly associated with AN and mood mapped to 25 genes (Supplementary Table 28). Twenty of the 29 loci jointly associated with AN and neuroticism mapped to 67 genes (Supplementary Table 29). Finally, 22 of the 36 loci jointly associated with AN and intelligence mapped to 76 genes (Supplementary Table 30).


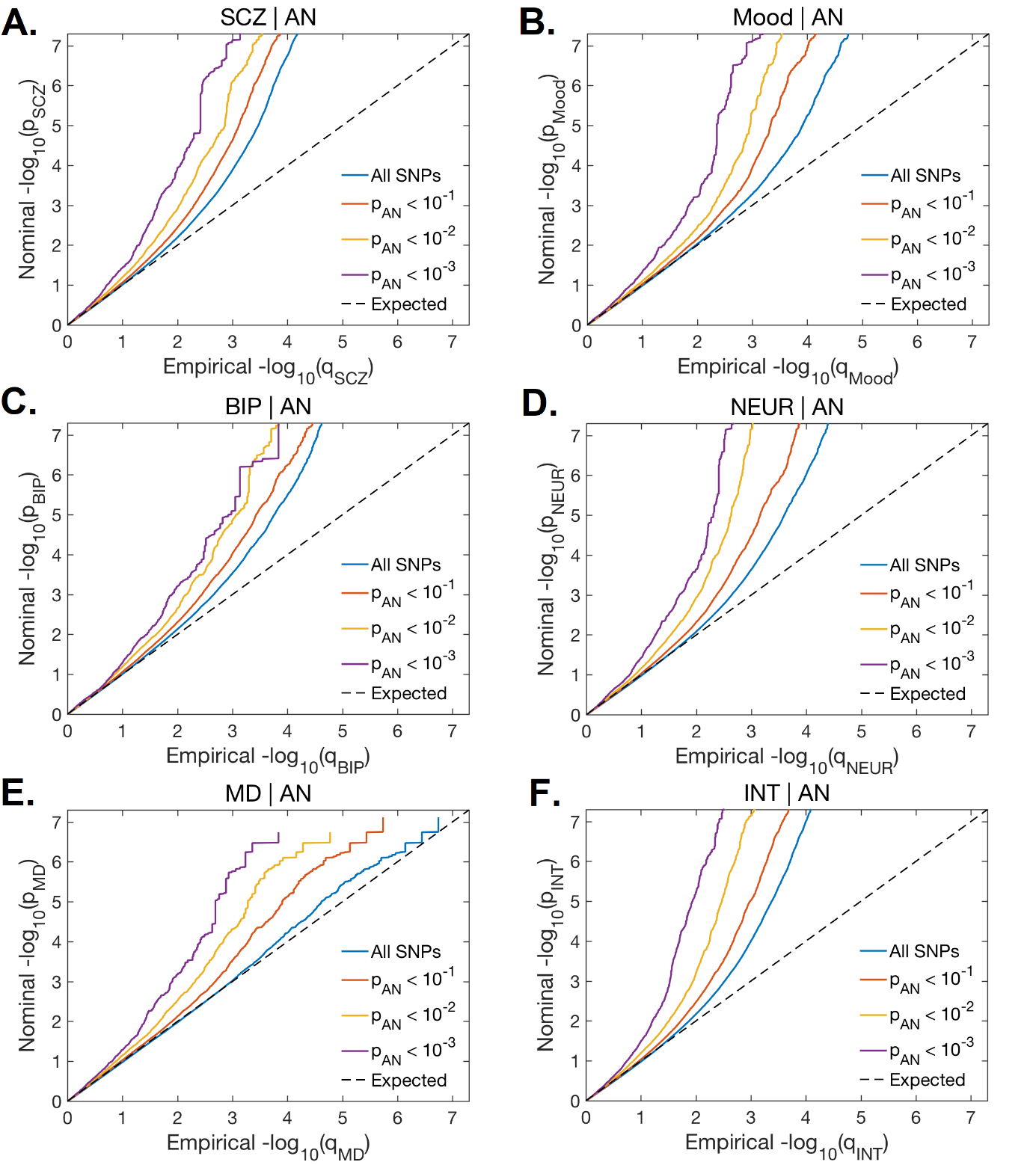


**Figure S1.** Panels A-F show conditional quantile-quantile plots of nominal vs. empirical schizophrenia (SCZ), bipolar disorder (BIP), major depression (MD), Mood, neuroticism (NEUR), and intelligence (INT) -log_10_ *p*-values as a function of the significance of the association with anorexia nervosa (AN) at the level of *p* < .10, *p* < .01 and *p* < .001. These show the quantiles of the observed *p*-values on the y-axis against the theoretical quantiles under no association on the x-axis. Deflections from the null line indicate systematic association.

**
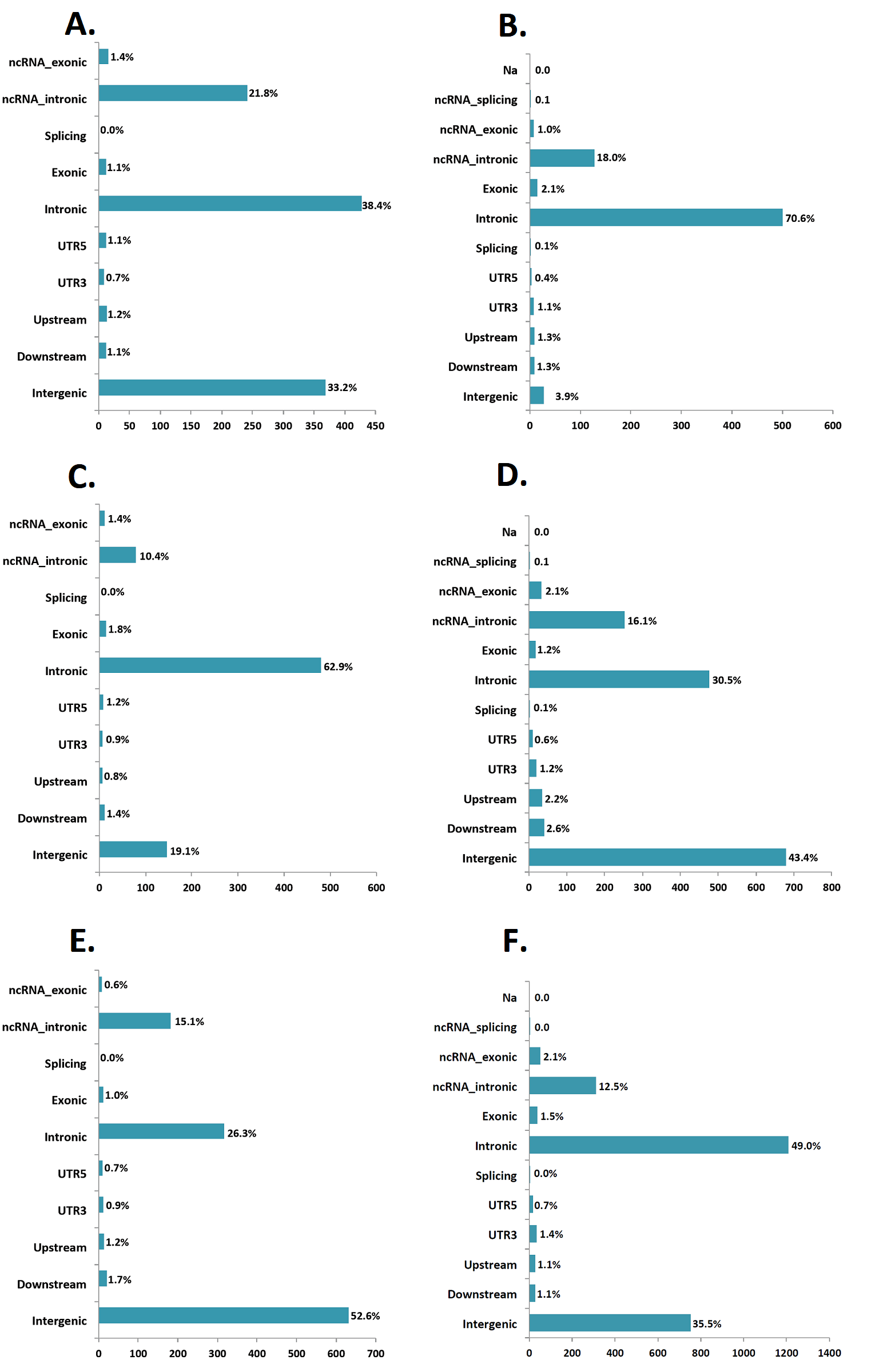
**

**Figure S2.** The distribution of functional annotation of all single-nucleotide polymorphisms in the significant genomic risk loci shared between anorexia nervosa and schizophrenia (A), mood (B), bipolar disorder (C), neuroticism (D), major depression (E), and intelligence (F).


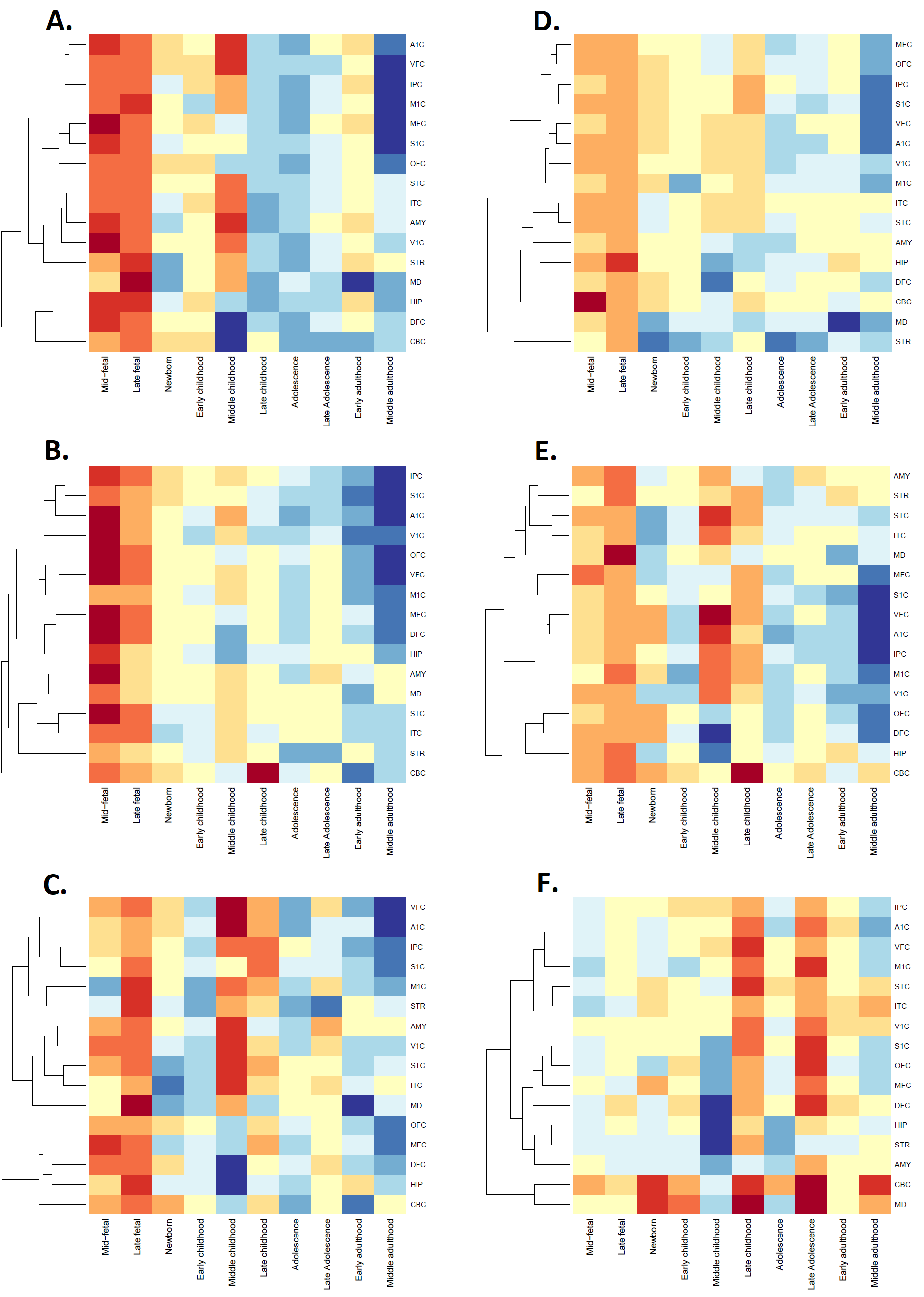


**Figure S3.** Dendrogram and heat-map showing spatiotemporal gene expression of all mapped genes jointly associated with anorexia nervosa and: schizophrenia (A), bipolar disorder (B), major depression (C), mood (D), neuroticism (E), and intelligence (F) using RNA sequencing data from BrainSpan over 11 developmental periods (columns) and 16 brain regions (rows). Gene expression is indicated from high (red) to low (blue). IPC = inferior parietal cortex, MFC = medial prefrontal cortex, STR = striatum, STC = superior temporal cortex, AMY = amygdala, CBC = cerebellum, HIP = hippocampus, DFC = dorsolateral prefrontal cortex, MD = medulla oblongata, OFC = orbital frontal cortex, V1C = primary visual cortex,M1C = primary motor cortex, A1C = primary auditory cortex, VFC = ventrolateral prefrontal cortex, S1C = primary somatosensory cortex, ITC = inferior temporal cortex.

**REFERENCES**

1. Ward J, Tunbridge EM, Sandor C, Lyall LM, Ferguson A, Strawbridge RJ, et al. The genomic basis of mood instability: identification of 46 loci in 363,705 UK Biobank participants, genetic correlation with psychiatric disorders, and association with gene expression and function. Mol Psychiatry. 2020 Nov;25(11):3091–9.

2. Nagel M, Jansen PR, Stringer S, Watanabe K, de Leeuw CA, Bryois J, et al. Meta-analysis of genome-wide association studies for neuroticism in 449,484 individuals identifies novel genetic loci and pathways. Nat Genet. 2018 Jul;50(7):920–7.

3. Savage JE, Jansen PR, Stringer S, Watanabe K, Bryois J, de Leeuw CA, et al. Genome-wide association meta-analysis in 269,867 individuals identifies new genetic and functional links to intelligence. Nat Genet. 2018 Jul;50(7):912–9.

4. Sudlow C, Gallacher J, Allen N, Beral V, Burton P, Danesh J, et al. UK biobank: an open access resource for identifying the causes of a wide range of complex diseases of middle and old age. PLoS Med. 2015 Mar;12(3):e1001779.

5. Mullins N, Forstner AJ, O’Connell KS, Coombes B, Coleman JRI, Qiao Z, et al. Genome-wide association study of more than 40,000 bipolar disorder cases provides new insights into the underlying biology. Nat Genet. 2021 Jun;53(6):817–29.

6. Pardiñas AF, Holmans P, Pocklington AJ, Escott-Price V, Ripke S, Carrera N, et al. Common schizophrenia alleles are enriched in mutation-intolerant genes and in regions under strong background selection. Nat Genet. 2018 Mar;50(3):381–9.

7. Watson HJ, Yilmaz Z, Thornton LM, Hübel C, Coleman JRI, Gaspar HA, et al. Genome-wide association study identifies eight risk loci and implicates metabo-psychiatric origins for anorexia nervosa. Nat Genet. 2019 Aug;51(8):1207–14.

8. Wray NR, Ripke S, Mattheisen M, Trzaskowski M, Byrne EM, Abdellaoui A, et al. Genome-wide association analyses identify 44 risk variants and refine the genetic architecture of major depression. Nat Genet. 2018 May;50(5):668–81.

9. Watanabe K, Taskesen E, van Bochoven A, Posthuma D. Functional mapping and annotation of genetic associations with FUMA. Nat Commun. 2017 Nov 28;8(1):1826.

10. Kircher M, Witten DM, Jain P, O’Roak BJ, Cooper GM, Shendure J. A general framework for estimating the relative pathogenicity of human genetic variants. Nat Genet. 2014 Mar;46(3):310–5.

11. Boyle AP, Hong EL, Hariharan M, Cheng Y, Schaub MA, Kasowski M, et al. Annotation of functional variation in personal genomes using RegulomeDB. Genome Res. 2012 Sep;22(9):1790–7.

12. Roadmap Epigenomics Consortium, Kundaje A, Meuleman W, Ernst J, Bilenky M, Yen A, et al. Integrative analysis of 111 reference human epigenomes. Nature. 2015 Feb 19;518(7539):317–30.

13. Zhu Z, Zhang F, Hu H, Bakshi A, Robinson MR, Powell JE, et al. Integration of summary data from GWAS and eQTL studies predicts complex trait gene targets. Nat Genet. 2016 May;48(5):481–7.

14. Colantuoni C, Lipska BK, Ye T, Hyde TM, Tao R, Leek JT, et al. Temporal dynamics and genetic control of transcription in the human prefrontal cortex. Nature. 2011 Oct 26;478(7370):519–23.

15. Johnson MB, Kawasawa YI, Mason CE, Krsnik Z, Coppola G, Bogdanović D, et al. Functional and evolutionary insights into human brain development through global transcriptome analysis. Neuron. 2009 May 28;62(4):494–509.

16. Kang HJ, Kawasawa YI, Cheng F, Zhu Y, Xu X, Li M, et al. Spatio-temporal transcriptome of the human brain. Nature. 2011 Oct 26;478(7370):483–9.

17. Kamburov A, Stelzl U, Lehrach H, Herwig R. The ConsensusPathDB interaction database: 2013 update. Nucleic Acids Res. 2013 Jan;41(Database issue):D793-800.
